# Dietary Polyphenols, Food Processing and Gut Microbiome: Recent Findings on Bioavailability, Bioactivity, and Gut Microbiome Interplay

**DOI:** 10.3390/antiox13101220

**Published:** 2024-10-10

**Authors:** Monika Sejbuk, Iwona Mirończuk-Chodakowska, Sercan Karav, Anna Maria Witkowska

**Affiliations:** 1 Department of Food Biotechnology, Medical University of Bialystok, Szpitalna 37, 15-295 Bialystok, Poland; iwona.mironczuk-chodakowska@umb.edu.pl (I.M.-C.); anna.witkowska@umb.edu.pl (A.M.W.); 2 Department of Molecular Biology and Genetics, Çanakkale Onsekiz Mart University, Çanakkale 17000, Türkiye; sercankarav@comu.edu.tr

**Keywords:** polyphenols, bioavailability, bioactivity, food processing, microbiome

## Abstract

Polyphenols are organic chemical compounds naturally present in plants, renowned for their anti-inflammatory, antioxidant, immunomodulatory, anticancer, and cardiovascular protective properties. Their bioactivity and bioavailability can vary widely depending on the methods of food processing and interactions with the gut microbiome. These factors can induce changes in polyphenols, affecting their ability to achieve their intended health benefits. Thus, it is essential to develop and apply food processing methods that optimize polyphenol content while maintaining their bioactivity and bioavailability. This review aims to explore how various food processing techniques affect the quantity, bioactivity, and bioavailability of polyphenols, as well as their interactions with the gut microbiome, which may ultimately determine their health effects.

## 1. Introduction

Polyphenols are naturally occurring organic compounds found in plants that are essential for human health. They are recognized for their anti-inflammatory, antioxidant, immunomodulatory, anticancer, and cardiovascular protective properties [1]. Their impact on health is primarily determined by their bioavailability, which is shaped by various food processing techniques and interactions with gut microbiota [2,3].

The gut microbiota, composed of millions of microorganisms within the digestive tract, is crucial in the metabolism of polyphenols, affecting their transformation and subsequent bioavailability [4,5]. The interactions between polyphenols and the microbiota can result in the formation of bioactive metabolites, which confer significant health benefits, including anti-inflammatory, antioxidant properties, and the protection of the gut barrier [6]. Thus, the relationship between polyphenols and gut microbiota is fundamental to human health.

In recent years, new insights have emerged regarding the interaction of polyphenols with the gut microbiota and the effects of various food processing techniques on the polyphenol content and bioavailability [3,7]. The available evidence suggests that processing techniques, including thermal treatment, drying, fermentation, and cold processing, can markedly alter the polyphenol content, consequently affecting their bioavailability and bioactivity [8,9]. Nevertheless, further extensive research is essential to enhance our comprehension of these processes and their implications for human health.

The objective of this review is to provide a comprehensive examination of the impact of diverse food processing techniques on the bioactivity and bioavailability of polyphenols, as well as their interactions with the gut microbiota. The objective of this review is to present updated information and perspectives on recent studies, which may offer new avenues for research on polyphenols and their health effects. This literature review is primarily concerned with studies conducted within the last five years. Nevertheless, some older studies of fundamental importance are also discussed.

## 2. Review

Polyphenols are organic chemical compounds that naturally occur in plants, constituting one of the most abundant and diverse groups of natural compounds in the plant kingdom. These compounds are classified as secondary metabolites, produced through the shikimate pathway derived from phenylpropanoids and/or the polyketide pathway [10]. With over 80,000 identified polyphenols, their molecular weights can reach up to 30,000 Da [10]. The polyphenol content in food products varies considerably, with certain items like dried herbs and cocoa powder containing over 1 g of polyphenols per 100 g of product [11,12]. In contrast, some plant-based foods, such as tomatoes, cucumbers, potatoes, and lemons, have significantly lower polyphenol content [12].

### 2.1. Classification of Polyphenols

**Figure 1:**
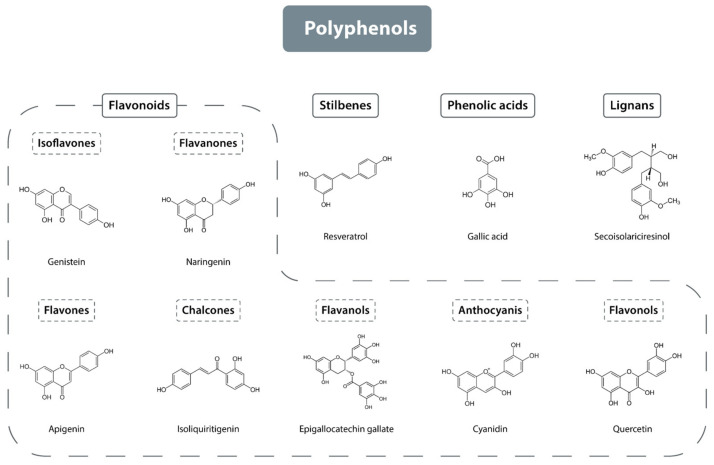
Classification of polyphenols.

Although polyphenols share common phenolic structural features, they exhibit significant structural diversity, resulting in their classification into several subgroups. Polyphenols can be categorized based on the number of phenol rings they contain and the structural elements that link these rings. The four primary structural groups of polyphenols are flavonoids, phenolic acids, lignans, and stilbenes (see Figure 1) [13,14,15]. Among these, flavonoids are the largest group, comprising approximately 60% of all polyphenols, followed by phenolic acids, which account for about 30% [14].

#### 2.1.1. Flavonoids

Flavonoids, a prominent subgroup within the polyphenol family, comprise over 6000 distinct chemical structures. The fundamental structure of flavonoids consists of two aromatic rings linked by three carbon atoms, forming an oxidized heterocyclic ring. Flavonoids can be categorized into two primary types based on the saturation of the central heterocyclic ring [16]. Saturated flavonoids include compounds such as flavanones, dihydroflavonols, and flavan-3-ols, while unsaturated flavonoids, characterized by a double bond between the 2 and 3 carbon atoms, include anthocyanidins, flavones, flavonols, and isoflavones. Chalcones represent a distinct subclass of flavonoids due to their unique chemical structure, characterized by an open-chain configuration instead of the closed heterocyclic ring typical of other flavonoids [17]. Although this structural classification is the most common, flavonoids can also be categorized based on molecular size and the degree of substitution on the A and B rings (flavonoids are composed of 15 carbon atoms arranged in three rings, labeled A, B, and C) [17].

Flavonoids in plants can exist either in free form or bound to sugars. Methylation of flavonoids can enhance their cellular uptake and stability, while glycosylation improves their solubility, metabolism, and distribution, as well as facilitating their transport across cell membranes [18].

Flavonoids are abundant in plants, nuts, and fruits, where they contribute to the fragrance, taste, and appearance of these foods. They serve various beneficial functions, including attracting pollinating insects, neutralizing reactive oxygen species, and acting as UV filters [19]. A diet rich in flavonoids is based on plant-based foods, including beverages such as beer, wine, and tea, as well as foods like fruits, vegetables, nuts, and edible flowers [20]. The phenolic compound content in plants is influenced by several factors, including the plant species, growth and storage conditions, temperature, soil type, environment, and harvesting methods [20].

Flavonoids are categorized into seven main classes based on their structural transformations: flavones, flavanones, isoflavones, flavonols, chalcones, flavanols, and anthocyanins (see Figure 1) [21,22]. Among these, flavones are one of the largest classes and are primarily found in parsley, red pepper, chamomile, celery, mint, and ginkgo [23]. Notable flavonols include quercetin, galangin, kaempferol, and myricetin, which are present in onions, asparagus, broccoli, lettuce, tomatoes, and apples [24]. Flavanones are predominantly found in citrus fruits [25], isoflavones are mainly in legume seeds [26], flavanols are present in fruits such as apples, pears, bananas, and blueberries [27]. Anthocyanins are found in various vegetables and fruits including blackcurrants, grapes, and berries, though they are not present in all fruits and vegetables, as their occurrence is linked to purple, red, and black pigmentation [28]. Chalcones occur in plants from the Fabaceae, Zingiberaceae, Moraceae, and Cannabaceae families [29].

#### 2.1.2. Phenolic Acids

Phenolic acids are generated as by-products from the degradation of cell wall polymers or through microbial activity, and are predominantly found in fruits, vegetables, grains, and seeds. They are primarily classified into two groups: derivatives of hydroxybenzoic acid and derivatives of cinnamic acid. Hydroxybenzoic acid derivatives include vanillic acid and gallic acid, whereas cinnamic acid derivatives encompass ferulic acid and caffeic acid [30].

#### 2.1.3. Lignans

Lignans are a diverse group of naturally occurring compounds synthesized via the shikimic acid pathway [31]. Their basic structure comprises two or more phenylpropanoid units. The monomers that contribute to lignan formation include cinnamic acid, cinnamyl alcohol, allylbenzene, and propenylbenzene [32]. Based on the molecular arrangement of these monomers, lignans are classified into two categories: classical lignans and neolignans. Neolignans are distinguished by their more varied structures compared to classical lignans [33]. To date, more than 200 classical lignans and 100 neolignans have been identified [34].

Lignans are present in a variety of plant-based products, including fruits, seeds, flowers, and leaves. While most lignans are found in their free form, some are bound to sugars, forming glycosides and other derivatives [33].

#### 2.1.4. Stilbenes

Stilbenes consist of two phenyl groups linked by a two-carbon methylene bridge. Most stilbenes are produced as a defensive response to injury or infection. They are predominantly synthesized in plants such as peanuts, grapes, rhubarb, and berries. A prominent compound in this group is resveratrol [35,36].

### 2.2. Bioavailability of Polyphenols

The impact of polyphenols on health is largely determined by their bioavailability. Bioavailability refers to the proportion of polyphenols that, after being released from the food matrix, is metabolized and absorbed, and subsequently reaches target cells or tissues to exert its bioactivity and produce beneficial health effects [2]. It is crucial to differentiate between bioavailability and bioactivity. Bioactivity pertains to the fraction of polyphenols that, after being released from the food matrix, reaches the gastrointestinal system, undergoes digestion, is absorbed by intestinal epithelial cells, and is subjected to metabolic changes in the intestines and liver [3,7]. This concept does not encompass the utilization of polyphenols in target cells or tissues, which is addressed by bioavailability [37]. Among polyphenols, isoflavones exhibit the highest bioavailability, followed by phenolic acids, flavanols, flavanones, flavonols, and anthocyanins. Conversely, the bioavailability of flavonoids tends to be relatively low due to their reduced bioactivity [38,39].

**Figure 2:**
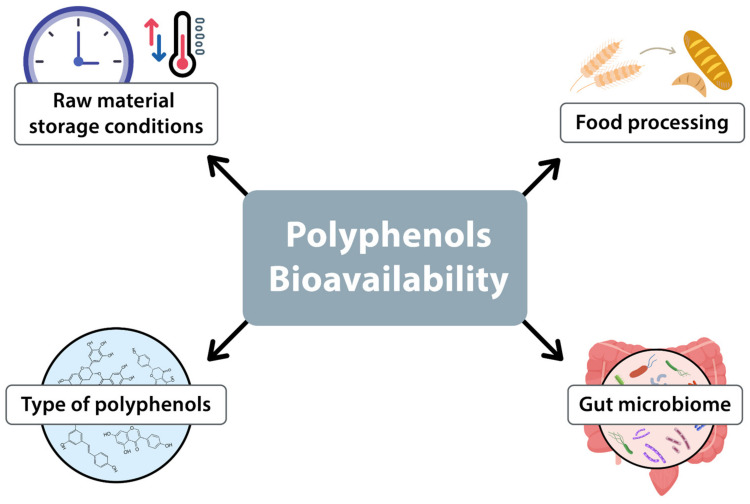
Factors influencing the bioavailability of polyphenols.

The bioavailability of polyphenols is influenced by several factors. Principal among these are the initial polyphenol content in the food, the processing methods employed, and the gut microbiota. The polyphenol content is also affected by the storage conditions of the raw material, including temperature, duration, and environmental factors. Furthermore, industrial and domestic processing methods, such as drying or fermentation, can alter the bioavailability of polyphenols in the final product (see Figure 2) [2,40].

The chemical structure of polyphenols plays a significant role in determining their bioavailability. In food, polyphenols are frequently present as polymers or glycosides. Glycosides are composed of an aglycone (the non-sugar component) and a glycone (the sugar component). Most polyphenols are resistant to adverse conditions during digestion and undergo hydrolysis primarily when exposed to intestinal enzymes or gut microbiota [41].

The bioavailability of polyphenols is regulated by both systemic and intestinal factors. Simple forms of polyphenols can be absorbed in the small intestine, while more complex forms reach the large intestine, where they are transformed into bioactive metabolites by the gut microbiota. Anthocyanins are unique in that they are absorbed directly from the stomach with the assistance of bilitranslocase, a specific membrane transporter [42,43,44].

In the intestine, polyphenols may interact with other dietary components, such as binding with macromolecules like dietary fiber. This interaction can lead to the formation of colloidal structures and chemical complexes, which may either enhance or reduce their bioavailability [45]. Polyphenol molecules that can be absorbed from the small intestine are typically aglycones. However, since most polyphenols in food are found as esters, glycosides, or polymers, they often reach the large intestine. There, they are transformed by the gut microbiota to facilitate their subsequent absorption [45].

### 2.3. Food Processing and Polyphenol Bioavailability

Food processing aims to extend the shelf life of products, enhance their taste and texture, and improve the bioavailability of active ingredients present in raw materials. However, some processing techniques can lead to a reduction in polyphenol content. Consequently, the final polyphenol content in a product is significantly influenced by the type of food processing methods used, the duration of processing, and the nature of the raw materials employed [8].

Among the various food processing methods, thermal treatment is one of the most commonly used. This includes processes such as boiling, steaming, frying, braising, baking, drying, sterilization, pasteurization, canning, roasting, and toasting. The impact of these methods on polyphenol content largely depends on the specific technique applied. High temperatures can either increase bioavailability by damaging plant cell walls or reduce polyphenol content through oxidation [9].

#### 2.3.1. Thermal Processing

Thermal processing is commonly employed in both domestic and industrial food preparation. Heat treatment leads to the breakdown of cell walls, which can facilitate the migration of polyphenolic compounds to different parts of the plant, potentially enhancing their bioavailability. However, this process also increases the susceptibility of polyphenols to oxidation. Furthermore, the thermostability of polyphenols varies, with some being more or less stable under heat [3,46].

Cooking, as a form of thermal processing, is often associated with significant losses of polyphenols, primarily because these compounds are water-soluble and can leach into the cooking liquid [46]. In contrast, cooking in oil tends to result in lower polyphenol losses [47]. The volume of the cooking solution also impacts polyphenol retention, with smaller amounts of water reducing the extent of polyphenol leaching [48]. Methods such as frying, steaming, and baking generally lead to lower losses of polyphenols compared to boiling [46].

Thermal processing methods such as cooking, grilling, and baking have been shown to reduce polyphenol content and diminish antioxidant activity in faba beans [49]. However, alternative processes such as cold and hot soaking, as well as sprouting for 3 to 5 days, may increase the phenolic content in faba beans. Despite this, thermal processing is more effective in reducing antinutritional factors compared to soaking and fermenting [49]. The impact of various thermal processing methods on polyphenol content is presented in Table 1.

The polyphenol content can vary depending on the thermal processing method and the type of raw material. Significant losses are generally observed during boiling, as polyphenols tend to leach into the cooking water. Furthermore, food processing techniques such as baking and frying may lead to higher levels of polyphenols, likely due to the breakdown of the food matrix. Careful selection of temperature and time can further improve polyphenol retention, emphasizing the importance of optimization.

#### 2.3.2. Drying

Drying is a process that reduces the water content in a product to a level that inhibits microbial growth and reproduction, thus preventing spoilage and extending shelf life. Various drying methods include freeze-drying, vacuum drying, sun drying, and air-drying [3].

Freeze-drying has been shown to be the most effective method for preserving polyphenol content in products, whereas high-temperature drying is generally associated with greater losses of these compounds [66]. However, it is not only the temperature, but also the duration of the drying process that influences polyphenol retention. The final polyphenol content in a product is significantly affected by the specific types of polyphenols present [3].

Moreover, the timing of harvest can influence both polyphenol levels and antioxidant activity. Extracts from olive leaves harvested in autumn and summer have been shown to contain higher levels of polyphenols, a trend that remains evident even after drying at high temperatures. This increase in polyphenol content is likely associated with higher solar radiation exposure during these seasons [67]. The impact of various drying methods on polyphenol content is presented in Table 2.

Drying can help preserve polyphenols in food, but it is essential to choose the appropriate temperature, duration, and processing method. Employing lower temperatures or freeze-drying appears to be effective, as these methods are associated with reduced polyphenol losses or even an increase in their content in the final product.

#### 2.3.3. Fermentation

Fermentation is a biological process in which microorganisms such as bacteria, yeast, and molds convert nutrients into metabolically active biomolecules. This important technology is widely used in food and beverage production, as well as in biotechnology, enhancing preservation, taste, texture, and nutritional value of products [85,86]. During fermentation, polyphenols can become more bioactive and bioavailable due to the action of enzymes involved in the process. Additionally, these polyphenols may support the growth of beneficial microbes while inhibiting harmful microorganisms [87,88].

During fermentation, microorganisms may degrade the glycosidic bonds of high-molecular-weight polyphenols, potentially influencing their direct utilization by intestinal microbes [87]. Additionally, enzymes associated with polyphenols, such as tannases, esterases, phenolic acid decarboxylases, and glycosidases, may be released during fermentation. These enzymes can liberate polyphenols that were previously bound within the food matrix, thereby enhancing their bioavailability and bioactivity [87,89]. Furthermore, fermentation can transform high-molecular-weight polyphenols into low-molecular-weight forms. This transformation often results from the enzymatic activity of microorganisms involved in the fermentation process. For instance, in the fermentation of pomegranate, ellagic tannins may be reduced to intermediate products like punicalin and gallagic acid, eventually yielding ellagic acid [90].

The fermentation process leads to changes in the composition and levels of polyphenols, which can impact their activity since different polyphenols exhibit varying levels of effectiveness. Fermentation typically converts high-molecular-weight polyphenols into smaller molecular forms, which are generally associated with higher antioxidant activity [87]. Fermented products often show increased bioactivity and bioavailability of polyphenols [91,92,93]. For instance, a study with human subjects revealed that consuming fermented soy altered isoflavone composition, which enhanced their absorption and increased the bioavailability of specific plasma metabolites [94].

The presence of polyphenols in a product can also positively impact the fermentation process by fostering the growth of beneficial microorganisms and suppressing pathogenic ones [95].

Fermenting foods can increase the polyphenol content in the final product and enhance their bioavailability and bioactivity. This process is associated with higher polyphenol levels due to the enzymatic breakdown of high-molecular-weight polyphenols. Additionally, the presence of polyphenols during fermentation can inhibit the growth of pathogenic microorganisms. The impact of various fermentation methods on polyphenol content is presented in Table 3.

Fermentation can significantly impact the polyphenol content in products, with critical factors including fermentation time, type of raw material, and the bacterial strains used. Selecting the appropriate fermentation duration is crucial for maximizing the benefits of polyphenols while minimizing their degradation. The type of raw material is also vital, as some materials contain higher levels of polyphenols that can be released or enhanced during fermentation. Additionally, the choice of bacterial strains, such as lactic acid bacteria, can enhance the bioavailability of polyphenols. Moreover, whether the fermentation process is controlled is an important consideration, as controlled conditions provide consistency that can help preserve or even increase polyphenol content.

#### 2.3.4. Cold Processing

Freezing is a method of cold food processing aimed at slowing down biochemical and physicochemical reactions, thereby extending the shelf life of products [113]. This technique generally has minimal impact on polyphenol content. For instance, freezing fresh raspberries at −30 °C did not alter their polyphenol levels. Additionally, freezing has been recognized as a highly promising initial processing method for improving the functional quality of black garlic [114]. Similarly, freezing organic butternut squash did not negatively affect its polyphenol content; rather, it was associated with an increase in bioaccessible polyphenols [115].

The speed of the freezing process can influence the polyphenol content of a product. Slow freezing tends to produce larger ice crystals, which can cause more damage to cell structures, while rapid freezing results in smaller ice crystals and potentially less cellular damage [116]. However, research on this topic is limited, and existing studies sometimes indicate that freezing may negatively affect polyphenol content and bioactivity. For example, apples frozen at −50 °C showed reduced levels of polyphenols and decreased antioxidant activity [117]. Thus, additional research is necessary to identify the optimal freezing temperatures and processing methods to maximize polyphenol content, bioavailability, and bioactivity in the final product. The impact of various cold processing methods on polyphenol content is presented in Table 4.

Cold processing methods can have a substantial impact on the polyphenol content in products. Generally, lower temperatures aid in preserving polyphenols by reducing enzymatic degradation and oxidation. However, the effects can vary based on the type of product and the specific polyphenols involved. In some instances, cold processing may enhance the bioavailability or concentration of polyphenols, while in others, it could result in minimal losses. Therefore, careful control of temperature and processing time is essential for optimizing polyphenol content in cold-treated products.

### 2.4. The Impact of Digestion on Polyphenol Content

For maintaining health and adequate nutrition, effective digestion and the breakdown of food into simpler constituents are essential [122]. During digestion, proteins are converted into amino acids, fats into fatty acids, and carbohydrates and glycerol into glucose. Additionally, other molecules, including plant metabolites, are transformed within the digestive tract according to their original structures and chemical properties [122,123]. Polyphenols in plant-based foods may be present in both free and bound forms. A crucial factor affecting their absorption is their release from the food matrix and dissolution during digestion [124]. The quantity of bioavailable polyphenols can differ markedly from their concentration in the food [125]. This disparity is largely influenced by the food matrix, interactions between polyphenols and other compounds, and the digestion process itself [126].

Chewing plays a crucial role in breaking down plant cell walls, which otherwise significantly impedes digestion. Saliva, which mixes with food during this process, can facilitate the dissolution of polyphenols present in fruits and fruit juices, potentially enhancing their bioavailability [127]. Additionally, proteins in human saliva may bind with certain polyphenols, thereby improving their absorption [128]. Hydrophobic polyphenols, in particular, have a greater tendency to interact with these proteins [126]. During the breakdown of plant cell walls, polyphenols may also bind to dietary fiber, creating a barrier that can affect their availability in the acidic environment of the stomach [129]. Despite this, such interactions can influence the fermentation of polyphenols by gut microbiota in the large intestine, leading to the production of metabolites with additional health benefits [129,130].

Most polyphenols remain stable during digestion in the mouth and stomach [131]. Their stability is attributed to their resistance to the conditions in these digestive stages, with hydrolysis primarily occurring through the action of intestinal enzymes and the gut microbiome [8]. Low-molecular-weight polyphenols can be directly absorbed in the small intestine; however, their absorption is limited to only 5–10%. In contrast, high-molecular-weight polyphenols are transported to the large intestine, where they are metabolized by the microbiota, allowing for their transformation into compounds with greater bioavailability [132].

In the intestine, polyphenols can interact with other substances, such as dietary fiber, which may influence their bioavailability [45]. Once absorbed, polyphenols and their metabolites are transported via the portal vein to the liver, where they undergo phase II metabolism, including methylation, sulfation, and glucuronidation [133]. This digestive process results in significant transformations of polyphenols and the formation of metabolites, which then reach target cells and exert their bioactivity, ultimately impacting human health [39].

### 2.5. Polyphenols and Their Interaction with the Gut Microbiome

The gut microbiome comprises millions of microorganisms residing in the digestive tract, playing a crucial role in sustaining human health by affecting digestion, metabolism, and the functionality of various bodily systems [4,5]. These microorganisms aid in breaking down undigested food, producing essential compounds such as vitamins and short-chain fatty acids, and preventing the colonization of harmful pathogens [5,134]. The composition of the gut microbiota is shaped by factors including genetics, medications, diet, age, and external conditions such as stress exposure [29,135,136].

**Figure 3:**
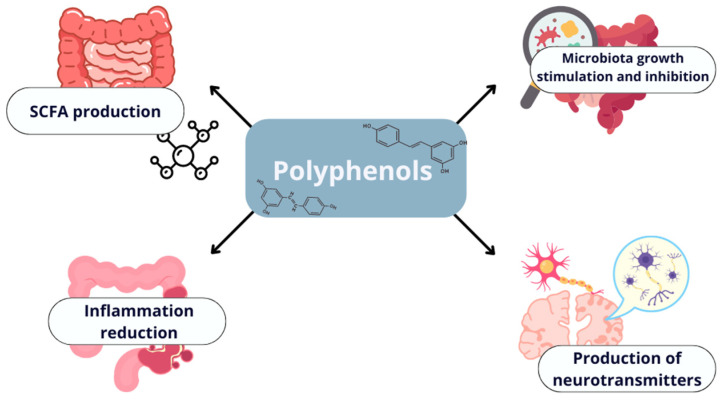
The impact of polyphenols on the gut microbiome.

Dietary polyphenols can significantly influence gut microorganisms by fostering beneficial alterations in their composition, thereby contributing to a healthier gut microbiota profile. These compounds can directly stimulate or inhibit the growth of specific bacterial strains [137]. Once metabolized by the gut microbiota, polyphenols are converted into metabolites with powerful antioxidant and anti-inflammatory properties, which support the production of short-chain fatty acids (SCFAs) and help maintain the integrity of the gut barrier. Furthermore, the microbiota play a role in suppressing intestinal inflammation and stimulating the production of neurotransmitters that influence the central nervous system [6]. One of the primary mechanisms by which polyphenols modulate the gut microbiome is through their prebiotic effect [138,139]. They can act as prebiotics by promoting the growth of beneficial bacteria, such as Lactobacillus and Bifidobacterium species, which in turn enhances the production of SCFAs like butyrate [140]. For example, catechins found in green tea are known to stimulate the growth of Bifidobacterium, leading to increased SCFA production, which is essential for maintaining intestinal health (see Figure 3) [141,142].

Gut microbiota dysbiosis can be induced by several factors, including an unhealthy diet, smoking, excessive alcohol consumption, and stress. This imbalance can result in reduced nutrient absorption, diminished production of SCFAs and vitamins, and may also lead to inflammation, thereby heightening the risk of gastrointestinal disorders and diseases [143]. However, the consumption of polyphenols may help regulate gut microbiome bacteria, potentially supporting overall gut health [144].

The combination of fresh grape pomace with a probiotic containing the Lactiplantibacillus plantarum strain may exhibit antimicrobial effects against *E. coli*, *Listeria monocytogenes*, and *Bacillus megaterium* [145]. Additionally, quercetin may enhance gut microbiome activity by modulating immune responses [146]. Metabolites derived from gallic acid are absorbed into the bloodstream, where they subsequently influence gut activity and potentially help reduce gut dysbiosis [147,148]. Polyphenols found in orange juice, such as hesperidin and naringin, promote the growth of beneficial bacteria like *Bifidobacterium* spp. and *Lactiplantibacillus* spp., which in turn increase SCFA production [149]. Similarly, polyphenols in cocoa exhibit positive effects on the gut microbiome; the metabolites produced from cocoa polyphenols stimulate the growth of beneficial bacteria such as *Lactobacillus* and *Bifidobacterium*, while inhibiting the growth of *Clostridium perfringens* [138].

The influence of polyphenols on the gut microbiome is particularly important in the context of metabolic diseases such as obesity [150,151,152] and type 2 diabetes [153,154,155,156]. Research has demonstrated that polyphenols can alter the composition of the gut microbiota by encouraging the growth of metabolically beneficial bacteria like *Akkermansia muciniphila*, which can enhance insulin sensitivity and reduce inflammation [157,158,159].

The metabolites derived from polyphenols can help reduce intestinal inflammation, promote the growth of beneficial microorganisms, inhibit the proliferation of pathogenic bacteria, and boost SCFA production. Therefore, maintaining a diet rich in plant-based foods high in polyphenols is essential for supporting gut health.

### 2.6. Strengths and Limitations

This article explores the relationship between dietary polyphenol content, the methods of their processing, and their impact on bioavailability and bioactivity. Additionally, it addresses the reciprocal interactions between polyphenols and the gut microbiota. The primary objective was to conduct an analysis of the current literature, synthesizing the information and methodologies that have informed previous research and contributed to the current understanding in this field.

One of the limitations of this review is its reliance on the existing literature, which unfortunately includes a limited number of in vivo studies specifically assessing the bioavailability and bioactivity of polyphenols. Additionally, there is a scarcity of data comparing polyphenol content in specific foods and dishes based on different processing methods. To draw more comprehensive conclusions, further research is needed to closely examine polyphenol content in processed products and to identify the most effective food processing methods.

Additionally, while this article reviews the current understanding of how food processing affects the bioactivity and bioavailability of polyphenols, as well as their interaction with the gut microbiome, more detailed studies are needed to further explore these topics. Such research would offer a more comprehensive and nuanced understanding of these complex interactions.

### 2.7. Summary

Polyphenols are organic compounds renowned for their wide-ranging health benefits, including anti-inflammatory, antioxidant, immunomodulatory, anticancer, and cardioprotective effects [1]. The efficacy of these compounds is substantially influenced by their bioavailability and bioactivity, which can be impacted by various factors such as the timing of harvest, soil type, sunlight exposure, and the processing methods applied to the raw material [3,7,37].

Thermal processing, including methods such as boiling, steaming, and baking, is among the most commonly used food processing techniques. Its impact on polyphenol content can vary; it may either preserve or degrade these compounds [3,46]. Drying, especially through freeze-drying, has been found to be effective in preserving polyphenols [3,66,67,68]. Fermentation, on the other hand, modifies food in ways that release polyphenols trapped within the matrix, enhancing their bioavailability and bioactivity [87,89]. Freezing typically does not significantly affect polyphenol content, though the results can vary depending on the specific temperatures used [116,160].

Proper digestion and the breakdown of food into simple components like amino acids, fatty acids, and glucose are essential for maintaining health and ensuring adequate nutrition [122]. Polyphenols, found in plant-based foods, can exist in either free or bound forms, with their bioavailability depending on their release from the food matrix during digestion [72]. Chewing and the presence of saliva can enhance the bioavailability of polyphenols, particularly those with hydrophobic characteristics, by facilitating their dissolution and binding to protein [126,127,128]. Most polyphenols remain stable during digestion in the mouth and stomach, with the majority of absorption occurring in the small intestine, although this process is somewhat limited [131,132]. In the intestines, polyphenols undergo further modifications by the gut microbiome, potentially leading to the formation of health-promoting metabolites [129,130].

During digestion, polyphenols are metabolized in the intestines, where the gut microbiome plays a crucial role in their transformation. This process generates metabolites such as phenolic acids, which may offer various health benefits, including anti-inflammatory effects and antioxidant properties [129,130].

Research into the relationships between gut microbiota, diet, health, and polyphenols is still in its early stages and continues to evolve [161]. A notable gap exists in studies examining how various food processing methods influence gut microbiota, highlighting a promising area for future investigation. Addressing this gap could deepen our understanding of how food processing techniques affect overall health.

### 2.8. Conclusions

Polyphenols in food offer a diverse range of health benefits, including anti-inflammatory, antioxidant, immunomodulatory, anticancer, and cardioprotective effects. However, their effectiveness is influenced by their bioavailability and bioactivity, which can be altered by various food processing methods. Additionally, the impact of polyphenols is closely linked to digestion and the gut microbiota. The relationship between polyphenols and the microbiota is reciprocal, with each component influencing and modulating the other.

Polyphenols can contribute to reducing inflammation in the gut, enhancing the production of short-chain fatty acids, and fostering the growth of beneficial microbes. Further research is necessary to better understand how different food processing techniques influence polyphenol content and quality, which will inform the selection of optimal processing methods. Equally important is a deeper investigation into the effects of polyphenols on the gut microbiome, which could lead to a more comprehensive understanding of their role in health maintenance.

The quantity of polyphenols present is largely dependent on the nature of the product being processed and the specific method employed. Appropriate processing techniques may result in minimal polyphenol reduction or even an increase in content, emphasizing the critical importance of method selection. Lyophilization and fermentation are notably effective approaches.

## Figures and Tables

**Table 1:** Recent case examples of variations in polyphenol levels due to thermal processing.

Thermal Processing	Outcome	Reference
Cooking fresh strawberries at 90 °C for 5 min	An increase in the content of flavan-3-ols/proanthocyanidins by 30%, catechin by 30%, epigallocatechin gallate by 45%, ellagic acid by 66%, and a decrease in the content of epicatechin by 16% and pelargonidin by 15%.	[50]
Steaming sorghum at 100 °C for 50 min	A decrease in total phenolic content, total flavonoid content, and condensed tannins (a 17% reduction in tannins).	[51]
Preparation of apple puree from apples	A 35% reduction in polyphenol content compared to a freeze-dried snack.	[52]
Boiling broccoli at 100 °C for 3 min	A 70% reduction in the content of phenolic acids and their derivatives, along with a 50% loss of phenolic compounds.	[53]
Chinese Kale at 97 ± 2 °C for 1, 2, and 3 min	An increase in total phenolic and flavonoid content. An increase in 2,2′-azino-bis(3-ethylbenzothiazoline-6-sulfonic acid) antioxidant activity assay and 2,2-diphenyl-1-picrylhydrazyl antioxidant activity assay.	[54]
Frying peanuts at 170 ± 3 °C for 1, 2, and 3 min	An increase in total phenolic and flavonoid content.	[55]
Cooking quince fruit purees at 120 °C for 20 min	An increase in the concentration of p-coumaric acid, trans-cinnamic acid, apigenin, and quercetin, and a decrease in the concentration of gallic acid.	[56]
Thermal processing of wheat and oat bran at 80 °C for 10 min	An increase in the total polyphenol content in wheat bran by 22.49%, while in oat bran it increased by 25.84%.	[57]
Blanching peppers in water and steam	A reduction in the total polyphenol content in steam-blanched pepper by 13.7% and by 10.9% in water-blanched pepper.	[58]
Ground rice hull underwent thermal processing in an autoclave at temperatures of 80, 100, 120, 130, and 140 °C for durations of 1, 3, and 5 h	The total flavonoid content in the sample heated for 1 h increased from 0.41 mg CE/g at room temperature (raw rice husk) to 1.43 mg CE/g at 130 °C, then slightly decreased to 1.42 mg CE/g at 140 °C. After 3 h of heating, the flavonoid content reached 1.43 mg CE/g at 140 °C, while after 5 h, it increased to 1.83 mg CE/g at 130 °C, then dropped to 1.42 mg CE/g at 140 °C. Total flavonoid content is expressed as mg catechin equivalents (CE)/g of dry matter.	[59]
Annual pepper at three distinct ripening stages (green, yellow, red) exposed to various thermal processing methods: baking, microwave-assisted baking, contact grilling, and steaming	Green pepper: an increase in polyphenol content during microwaving and baking (from 2.75 to 15.24 mg GAE/g dry basis). For steaming (from 2.75 to 6.25 mg GAE/g d.b.). For the ‘Ożarowska’ variety, microwaving (from 7.40 to 20.18 GAE µM/g d.b.), and boiling (from 2.75 to 9.76 GAE µM/g d.b.). Yellow pepper: a reduction in polyphenol content was observed during all heat treatment methods. The largest reduction occurred after steaming (from 17.00 to 6.31 mg GAE/g d.b.). Baking resulted in a 47% reduction (from 17.00 to 9.02 mg GAE/g d.b.). Red pepper: reduction in total polyphenol content during simultaneous microwaving and baking (from 17.26 to 5.46 mg GAE/g d.b.) and during steaming (from 17.26 to 6.01 mg GAE/g d.b.). Grilling led to a 36% increase in total polyphenol content (from 17.26 to 23.56 mg GAE/g d.b.). In a previous study, microwaving resulted in an 11% reduction in total polyphenol content (15.37 GAE µM/g d.b.), and boiling in water led to more than a ninefold reduction (from 15.37 to 1.86 GAE µM/g d.b.).	[60]
Thermal pasteurization and high-pressure processing of orange juices	Thermal pasteurization: reduction in flavonoid and anthocyanin content. High-pressure processing: increase in flavonoid and anthocyanin content.	[61]
Processing of radish at three different temperatures (70, 80, and 90 °C) with 90% relative humidity	The phenol content decreased from 3.37 mg/g dry weight to 1.88 and 2.92 mg/g DW within the first 3 days at a temperature of 70 °C. The flavonoid content in radish increased steadily at 70 °C and 80 °C, rising from 0.76 mg/g DW. to 1.82 and 2.08 mg/g DW, respectively.	[62]
Lord variety potatoes subjected to different thermal processing methods: boiling, steaming, steaming in a RETIGO convection-steam oven, microwaving, and grilling	Potatoes: the total polyphenol content in raw potatoes was 183.5 mg total polyphenols, expressed as gallic acid equivalents (GAE) per 100 g of dry matter. The highest polyphenol content was found in grilled potatoes (318.5 mg/100 g dry matter), while the lowest was in boiled potatoes (210.9 mg/100 g dry matter).	[63]
Yellow-skinned and red-skinned onions subjected to boiling, followed by thermal processing such as baking, boiling, and frying.	Boiling onions resulted in a decrease in total phenolic content, with a 37.5% reduction in yellow-skinned onions and a 13.9% reduction in red-skinned onions. Frying: caused the largest increase in total phenolic content (61.6% in yellow-skinned onions and 35.1% in red-skinned onions). Baking: led to a 58.7% increase in yellow-skinned onions, while grilling resulted in a 30.1% increase in red-skinned onion.	[64]
Raw sprouts subjected to baking at different temperature ranges	Total polyphenol content is comparable in baked products and raw sprouts.	[65]

**Table 2:** Recent examples of polyphenol alterations during drying processes.

Drying Methods	Outcome	Reference
Drying of orange-black tea using a box dryer under the following conditions: 45 °C for 2 h, 50 °C for 6 h, 80 °C for 10 h, 65 °C for 2 h, and 75 °C for 15 min, as well as continuous drying for 4 days under sunlight at daytime temperatures of 35–40 °C and storage at 4 °C at night	Sun drying of orange-black tea leads to greater polyphenol loss compared to hot air-drying techniques. Lower temperatures result in better polyphenol retention compared to the use of higher temperatures. The best polyphenol retention values were observed in tea dried with hot air at low temperatures.	[68]
Mangoes and pineapples dried in direct sunlight, dried in sunlight shaded by white fabric, dried in sunlight shaded by black fabric, and dried in a conventional solar dryer	Reduction in total phenolic content compared to fresh fruit. Total phenolic content in mango (g/100 g): - Fresh 0.85 ± 0.02 - Drying in direct sunlight 0.44 ± 0.06 - White-cloth shade 0.46 ± 0.04 - Black-cloth shade 0.51 ± 0.04 Total phenolic content in pineapple (g/100 g): - Fresh 0.43 ± 0.02 - Drying in direct sunlight 0.30 ± 0.04 - white-cloth shade 0.32 ± 0.03 - Black-cloth shade 0.31 ± 0.02 - Conventional solar dryer 0.39 ± 0.02	[69]
Total polyphenol content in chamomile extract obtained through different drying methods	Convective oven drying resulted in the highest polyphenol content (56.94 ± 5.98 mg GAE/g powder), while the lowest polyphenol content was observed with spray drying at 140 °C and a flow rate of 10.5 mL/min (42.79 ± 3.98 mg GAE/g).	[70]
Comparison of polyphenol content in fresh lychee versus lychee dried using freeze-drying, vacuum drying, and oven drying methods	Fresh fruits exhibited higher polyphenol content compared to all drying methods.	[71]
Wild blueberries dried at 90 °C and freeze-dried	An increase in total polyphenol and flavonoid content compared to raw wild blueberries.	[72]
Home drying of raw apples at 50 °C and 70 °C	A decrease in total polyphenol content after drying at both temperatures. The total polyphenol content (mg GAE/100 g dry weight) in fresh fruits was 165.3 ± 6.4, while in fruits dried at 50 °C, it was 121.4 ± 13, and in those dried at 70 °C, it was 81.6 ± 2.2.	[73]
Comparison of citrus peel drying in an oven at 40 °C versus vacuum drying at 60 °C	Oven drying at 40 °C was associated with higher total polyphenol content, hesperidin, and rutin in the lemon peel extracts, while vacuum drying at 60 °C was associated with better retention of hesperidin, p-coumaric, and trans-ferulic acids in clementine peel extracts.	[74]
Three varieties of lentils (French green whole seeds, green whole lentil seeds, and red lentil seeds without a seed coat) were dried at 60, 80, and 100 °C until the moisture content was reduced below 10%	An increase in total flavonoid content across all varieties.	[75]
Bulgur grain was cooked at 100 °C for 42–53 min, then dried at 60 °C for 180 min	Drying at 60 °C resulted in a reduction of total polyphenol content from 0.57 ± 3.20 × 10^−5^ to 0.62 ± 5.5 × 10^−4^ (mg GAE/g DM) and a reduction of total flavonoid content from 0.54 ± 3.46 × 10^−4^ to 0.64 ± 1.9 × 10^−5^ (mg GAE/g DM).	[76]
Drying chestnuts for chestnut flour using a traditional “metato” dryer at an average temperature of 37 °C, in a laboratory oven at 70 °C, and with an air dehumidifier at 40 °C	Higher polyphenol content in air-dried chestnut flours compared to other drying methods.	[77]
Fresh mulberries dried with hot air at 60 °C for 48 h or completely frozen in a deep freezer at −50 °C for 24 h, then freeze-dried under vacuum for 48 h at −50 °C	Significantly higher total flavonoid, phenol, and neochlorogenic acid content after freeze-drying compared to hot air-dried fruits.	[78]
Sun drying of pistachios (*Pistacia vera* L.)	Reduction of compounds with high antioxidant activity, such as anthocyanins and trans-resveratrol, due to sun drying. The flavonoid content in mature grains was 63.7 mg/100 g dry matter, while after the drying process, it was 24.3 mg/100 g dry matter.	[79]
Drying two varieties of olive leaves (*Istrska belica* and *Leccino*) using air at room temperature in a dark place, air-drying at 105 °C for 90 min, and freeze-drying by sublimation	The highest oleuropein content (13.60 mg/g dry leaves) in *Istrska belica* was obtained through freeze-drying. For *Leccino*, the highest oleuropein content was achieved in air-dried leaves at room temperature, followed by freeze-drying and air-drying at 105 °C. The oleuropein content in the *Leccino* variety is significantly lower than in *Istrska belica*.	[80]
Drying of date plums at four temperatures (50, 60, 70, and 80 °C) and three air velocities (0.5, 1.0, and 1.5 m/s)	The total phenol and flavonoid content in dried fruits increased as the temperature rose from 50 °C to 70 °C.	[81]
Freeze-drying and air-drying of green peas and green beans	An increase in bioactive compound content.	[82]
Roasting cocoa beans	Polyphenol content increased in roasted cocoa beans compared to unroasted cocoa beans.	[83]
Drying coffee fruits using four methods: microwave vacuum drying, room temperature drying (at 20 to 30 °C), heat pump drying (at 40 or 50 °C), and freeze-drying	An increase in total phenol content in coffee dried by microwave vacuum drying, heat pump drying, and freeze-drying.	[84]

**Table 3:** Recent examples of variations in polyphenol content during fermentation.

Fermentation	Outcome	Reference
Fermentation of avocado leaves with lactic acid bacteria strains	An increase in total polyphenol content after fermentation with most strains (except *L. plantarum CECT 748T*).	[96]
Lactic acid fermentation of legume seeds	An increase in the number of lactic acid bacteria and isoflavones, along with a reduction or complete elimination of pathogenic microorganisms.	[97]
Spontaneous fermentation of black wolfberry	An increase in total polyphenol (by 311.34%) and flavonoid (by 42.91%) content, along with a decrease in total anthocyanin (by 5.63%) content.	[98]
Inoculated lactic acid fermentation in wheat sourdough	Content of polyphenolic compounds (mg/100 g dry matter) after 72 h of fermentation: - unfermented 208.30 - spontaneous fermentation 262.35 - *Lactobacillus casei* fermentation 295.96 - *Lactobacillus plantarum* fermentation 309.59	[99]
Fermentation of artichoke by-products (bracts, stems, and leaves)	An increase in the content of individual studied flavonoids and a decrease in chlorogenic acid content.	[100]
Fermentation of sucrose-sweetened and unsweetened ginger juice	An increase in total polyphenol and flavonoid content. Higher polyphenol content in the sucrose-sweetened ginger juice sample (from 10.82 to 18.3 mg GAE/g dry matter) compared to the unsweetened ginger juice (from 10.18 to 14.9 mg GAE/g dry matter).	[101]
Fermentation of spelt flour	An increase in total polyphenol content in fermented spelt flour (3.65 ± 0.17 mg GAE/g dry matter) compared to unfermented flour (0.99 ± 0.08 mg GAE/g dry matter).	[102]
Fermentation of black tea using a kombucha starter culture	An increase in total polyphenol content.	[103]
Fermentation of tomato purees	An increase in total polyphenol content, with a decrease in aglycone-polyphenols compared to raw tomato purees.	[104]
Spontaneous fermentation of green, yellow, and red peppers	An increase in total polyphenol content.	[105]
Fermentation of fresh Fuji apples with activated lactic acid bacteria	An increase in total polyphenol content compared to fresh Fuji apples (fresh apple: 4.27 ± 0.16 mg GAE/g DM, apple after 25 days of fermentation: 6.22 ± 0.21 mg GAE/g DM). A decrease in the content of 12 analyzed polyphenolic compounds compared to fresh Fuji apples.	[106]
Controlled fermentation of three kale varieties (Nero di Toscana, Scarlet, and Halbhoner Grüner Krauser)	Increased bioavailability of phenolic compounds, along with an increase in total polyphenol content and antioxidant activity in fermented products.	[107]
Controlled fermentation of blueberry pomace using *Lactobacillus acidophilus* (LA), *Lactobacillus plantarum* (LP), *Lactobacillus casei* (LC), *Aspergillus oryzae* (AO), *Aspergillus niger* (AN), and *Monascus anka* (MA)	The highest polyphenol content was observed after fermentation with AN (209.18 ± 7.60 mg gallic acid equivalents/g dry weight) compared to unfermented pomace (167.67 ± 7.95 mg GAE/g DW). The highest total polyphenol content was recorded after fermentation with LA (93.71 ± 2.07 mg rutin equivalents/g DW) compared to unfermented pomace (43.51 ± 2.39 mg RE/g DW). The highest anthocyanin content was found after fermentation with LA (7.81 ± 0.53 mg cyanidin-3-glucoside equivalent per gram of pomace (mg C3G/g DW)), although there was a decrease compared to unfermented pomace (8.94 ± 0.56 mg C3G/g DW).	[108]
Controlled fermentation of ground white and red quinoa seeds	An increase in total polyphenol content by over 50%.	[109]
Solid-state fermentation of lentils with an edible fungus (*Pleurotus ostreatus*)	An increase in total polyphenol content compared to the unfermented product (from 2.1 to 3.2 mg gallic acid equivalent per g dry matter).	[110]
Controlled fermentation of multifloral bee pollen	An increase in total polyphenol content.	[111]
Fermentation of eight different pigmented sorghum varieties	An increase in total polyphenol content.	[112]

**Table 4:** Examples of alterations in polyphenol content attributed to cold processing.

Cold Processing	Outcome	Reference
Storage of sliced organic butternut squash (*Cucurbita moschata*) at −80 °C	An increase in polyphenol bioavailability.	[115]
Storing apples and strawberries for 12 months at −20 °C and 4 °C	No significant differences in proanthocyanidin concentration. Minimal reduction in total polyphenol content.	[118]
Strawberries stored at 0 °C	An increase in total polyphenol content.	[119]
Freezing arugula leaves, spinach, and watercress by-products at −20 °C	An increase in total polyphenol content in frozen spinach leaves compared to fresh leaves (frozen: 1065.35 ± 50.68 mg GAE per 100 g dry mass; fresh: 474.14 ± 8.19 mg GAE per 100 g DM). An increase in total polyphenol content in frozen arugula leaves (frozen: 1186.65 ± 34.44 mg GAE per 100 g DM; fresh: 469.51 ± 34.14 mg GAE per 100 g DM). An increase in total polyphenol content in watercress by-products (frozen: 1262.13 ± 51.13 mg GAE per 100 g DM; fresh: 629.60 ± 24.94 mg GAE per 100 g DM).	[120]
Germination of flat kale (*Brassica oleracea* var. *acephala*) at different temperature ranges (21, 8, and −8 °C)	A decrease in total phenol content in sprouts exposed to −8 °C (10.54 ± 0.57 µg GAE mg^−1^ dry weight) compared to sprouts grown at 21 °C (13.17 ± 0.41 µg GAE mg^−1^ DW) and 8 °C (13.43 ± 1.02 µg GAE mg^−1^ DW). A similar decrease in total flavonoid content at 8 °C and −8 °C compared to the control group (3.73 ± 0.21 µg CE mg^−1^ DW). However, low temperatures led to an increase in total phenolic acid content.	[121]
